# A Novel Adversarial Example Detection Method Based on Frequency Domain Reconstruction for Image Sensors

**DOI:** 10.3390/s24175507

**Published:** 2024-08-25

**Authors:** Shuaina Huang, Zhiyong Zhang, Bin Song

**Affiliations:** 1 Information Engineering College, Henan University of Science and Technology, Luoyang 471023, China; 205400000024@stu.haust.edu.cn (S.H.); songbin@haust.edu.cn (B.S.); 2 Henan International Joint Laboratory of Cyberspace Security Applications, Henan University of Science and Technology, Luoyang 471023, China; 3 Henan Intelligent Manufacturing Big Data Development Innovation Laboratory, Henan University of Science and Technology, Luoyang 471023, China

**Keywords:** adversarial detection, deep learning attacks, frequency domain, gradient masking, reconstruction

## Abstract

Convolutional neural networks (CNNs) have been extensively used in numerous remote sensing image detection tasks owing to their exceptional performance. Nevertheless, CNNs are often vulnerable to adversarial examples, limiting the uses in different safety-critical scenarios. Recently, how to efficiently detect adversarial examples and improve the robustness of CNNs has drawn considerable focus. The existing adversarial example detection methods require modifying CNNs, which not only affects the model performance but also greatly enhances training cost. With the purpose of solving these problems, this study proposes a detection algorithm for adversarial examples that does not need modification of the CNN models and can simultaneously retain the classification accuracy of normal examples. Specifically, we design a method to detect adversarial examples using frequency domain reconstruction. After converting the input adversarial examples into the frequency domain by Fourier transform, the adversarial disturbance from adversarial attacks can be eliminated by modifying the frequency of the example. The inverse Fourier transform is then used to maximize the recovery of the original example. Firstly, we train a CNN to reconstruct input examples. Then, we insert Fourier transform, convolution operation, and inverse Fourier transform into the features of the input examples to automatically filter out adversarial frequencies. We refer to our proposed method as FDR (frequency domain reconstruction), which removes adversarial interference by converting input samples into frequency and reconstructing them back into the spatial domain to restore the image. In addition, we also introduce gradient masking into the proposed FDR method to enhance the detection accuracy of the model for complex adversarial examples. We conduct extensive experiments on five mainstream adversarial attacks on three benchmark datasets, and the experimental results show that FDR can outperform state-of-the-art solutions in detecting adversarial examples. Additionally, FDR does not require any modifications to the detector and can be integrated with other adversarial example detection methods to be installed in sensing devices to ensure detection safety.

## 1. Introduction

Deep learning techniques [1,2], have achieved significant success in multiple fields including computer vision [3], autonomous driving [4], motion tracking [5], and speech recognition [6]. However, Szegedy et al. [7] have identified that these networks are susceptible to adversarial examples (AEs), which poses a substantial barrier to their broader adoption. Adversarial examples are created when an attacker introduces subtle, carefully crafted perturbations to a correctly classified image, leading the CNNs to classify it incorrectly with high confidence. With the widespread adoption of deep learning technology, developing effective defenses against such adversarial attacks is vital for enhancing the security of neural network models [8,9].

As CNNs can be susceptible to adversarial attacks, extensive research has been conducted on such attacks [10,11,12,13]. These attack methods include FGSM [10], the Carlini and Wagner (C&W) [11] method, the Basic Iterative Method (BIM) [12], etc. Given that adversarial attacks typically involve the addition of perturbations to the input, causing misclassification errors in deep learning models, strategic input transformations and model reconstructions can serve as defenses against these attacks [14,15,16].

Adversarial defense strategies can be primarily classified into two categories: model-based defenses [17,18], and input sample-based defenses [19,20]. Model-based defenses modify the parameters of CNNs for better robustness. Employed techniques include adversarial training [21], gradient regularization [22], and model distillation [23]. To accommodate model adaptation to sample sets of varied attributes, Goodfellow et al. [10] proposed adversarial training, wherein both adversarial and normal samples are applied in model training to improve classification accuracy. Adversarial trained DNNS identify clean examples with much less accuracy than normally trained DNNS, limiting their large-scale use in real-world scenarios. To deal with the existing problems, Papernot et al. [23] obtained a distillation model with similar architecture to the original model by using input samples and probability distribution training. The method trains the teacher network at a distillation temperature of T to acquire prior knowledge. This prior knowledge is then transferred to the student network, which is also trained at the same distillation temperature T, to produce the final output. The defensive distillation approach enhances model robustness without requiring smoothing of the neural network’s output. Input sample-based defense involves designing algorithms to remove perturbations or to detect adversarial examples by analyzing their characteristics. Techniques in this domain include data feature squeezing [24], adversarial feature analysis [25,26], and input reconstruction [27]. While model-based defense techniques bolster model robustness, they may fail to recognize adversarial samples and potentially diminish accuracy on standard inputs. Defense based on input samples does not affect the classification accuracy of the model on normal samples. After adversarial examples are detected, we can perform pre-processing operations such as denoising to disable the disturbance and make the defense more fine-grained.

Although the current detection methods have made progress, there are still problems of poor detection performance. For instance, setting thresholds for methods that rely on data feature squeezing involves considerable uncertainty, leading to significant deviations in practical applications. Utilizing the Conditional Pixel CNN [28] model, multiple input images are reconstructed to discern differences and detect adversarial samples. Although this model demonstrates robust generalization capabilities, the image generation process, which sequentially constructs each pixel, results in prolonged training and inference times. Moreover, detection strategies based on Local Intrinsic Dimensionality (LID) [29] necessitate an acute understanding of the targeted model’s hyperparameters and structures, which can be challenging to ascertain. Such strategies also exhibit limited efficacy in identifying adversarial examples produced by alternative attack models.

To overcome the existing issues, this study attempts to introduce a novel detection method based on frequency domain reconfiguration to effectively prevent the most advanced adversarial attacks. Our approach innovatively utilizes frequency domain analysis as a distinct measure. We observe that most adversarial perturbations manifest as high-frequency signals or are superimposed onto high-frequency components. Neural networks, during training, tend to overfit to low-frequency signals, making them vulnerable to being deceived by the high-frequency signals within adversarial samples. To counteract this, we apply neural networks in conjunction with discrete Fourier transform to decompose the input image into its frequency domain components, followed by convolutional filtering to attenuate noise. Subsequently, we reconstruct the denoised samples back into the spatial domain to restore the image and eliminate adversarial distortions. Additionally, we incorporate gradient masking to strengthen the intrinsic link between prediction confidence and the authenticity of the original image. Finally, a consistency check is implemented to identify AEs. In summary, the following contributions are presented:

- This study indicates that adversarial examples are easily eliminated in the frequency domain, while normal examples are often immune to this operation in the frequency domain, providing a novel research direction for AE detection.
- This study proposes a new detection method that distinguishes AEs from normal samples through training a frequency domain reconstructor. Specifically, we transform adversarial examples into the frequency domain to eliminate adversarial attacks and restore images.
- Gradient masking is introduced into the proposed FDR method. This method not only strengthens the internal relationship between the prediction confidence and the original image authenticity but also enhances the recognition accuracy for complex examples.

## 2. Related Work

The main work of this paper is to improve the detection accuracy of AEs to better identify AEs. Therefore, the first part of this section introduces the research status in the field of generating AEs. The current popular adversarial attack algorithms are analyzed and reviewed. We use these attack algorithms to generate adversarial examples for detection. The second part analyzes and summarizes the methods of adversarial example detection from recent years, and identifies key problems to be overcome.

### 2.1. Adversarial Examples Generation

Szegedy et al. [7] first found that convolutional neural networks have blind spots in recognizing adversarial examples. This refers to inputs that have been perturbed in a subtle manner, imperceptible to humans but leading a target model to make high-confidence incorrect predictions. Researchers refer to the process of generating such subtle perturbations as adversarial attacks. The images with perturbations produced by adversarial attacks are referred to as adversarial examples (AEs) [30]. Based on the malicious person’s knowledge of the target network’s architecture or parameters, adversarial attacks are divided into two types: white-box and black-box. White-box attacks require the participant to have a prior knowledge of the target network, including its hidden layer, hyperparameters, gradient information and outputs from each layer. The principal methodologies for implementing such attacks include optimization-based attacks, gradient-based attacks, and generation-based attacks. Black-box attacks are conducted without detailed knowledge of the target network. Attackers usually indirectly obtain model information based on output labels and confidence levels or use the transferability AEs to launch attacks. The primary implementation algorithms are transfer-based attacks and query-based attacks. This section introduces several representative adversarial attack methods. The AEs generated by these methods will be used in this study for experimental testing.

Goodfellow et al. [10] initially put forward a target-free attack algorithm called the fast gradient symbol method (FGSM), generating AEs under the L∞ norm limit of original clean samples. The principle of FGSM is to add disturbance that causes maximum loss to the model along the opposite direction of the normal example gradient, thus reducing the classification confidence. FGSM represents a one-step attack process conducted within a white-box setting. The gradient of the classification model regarding input *x* is calculated. The attack direction is determined with the use of a symbolic function, which is then scaled by a specified step to compute the adversarial perturbation. This perturbation is supplemented to the initial input *x* to obtain adversarial sample $\hat{x}$ under the FGSM attack. FGSM can generate adversarial examples quickly, but the attack success rate of FGSM depends on the selected hyperparameters. The generated AEs can be written as the following:

(1) $$\hat{x}=x+\alpha \cdot sign\left(\nabla_{x}L\left(x,y\right)\right)$$

The FGSM method does not require altering the network’s model structure or specific pixels in the input sample. Instead, it focuses on the direction of the gradient perturbations, calculating the gradient of each input image to modify its structure.

Carlini and Wagner [11] put forward the C&W attack method, which takes into account both high attack accuracy and low disturbance. The algorithm optimizes perturbations by limiting the L0, L2, or L∞ norms, and its objective function consists of optimization problems that maximize loss functions and minimize perturbations. In the C&W algorithm, AEs can be acquired by addressing the following optimization problems:

(2) $$\left\{\begin{array}{c} \min D(x,t)+c\cdot f(t)\\ t=x+\delta \end{array}\right.\;\;s.t.\;t\in {\left[0,1\right]}^{n}$$

where D(·) is a function of the distance measurement, *x* is the normal sample, *t* is the adversarial sample, and δ indicates the disturbance added for each iteration. Parameter *c* can control the balance between the added disturbance value and the confidence of the wrong classification. f(·) is the design objective function:

(3) $$f\left(x^{\prime }\right)=\max\left(\max\left\{Z{\left(x^{\prime }\right)}_{i}:i\neq t\right\}-Z{\left(x^{\prime }\right)}_{t},-k\right)$$

where Z(·) is the logical value of the previous layer of Softmax, *i* is the label category, *t* is the label category of target attack, and parameter *k* can control the confidence that the adversarial sample is misjudged. The larger the *k* value, the higher the success rate of the adversarial attack. The proposed method has a good attack effect but a high cost.

Kurakin et al. [12] introduced the BIM method, which improves the performance of FGSM through multiple iterations with small step sizes. BIM follows the gradient direction of the input loss function, performs FGSM in smaller steps, and trims the updated adversarial example into an effective range. Then, the AEs generated by BIM method can be described as follows:

(4) $$x_{i+1}=Clip_{\varepsilon }\left\{\begin{array}{c} x_{i}+\alpha \cdot sign\left(\nabla_{x}L\left(x,y\right)\right) \end{array}\right\}$$

where *i* is the number of iterations, and Clip is a clipping function that limits the perturbation to the ε neighborhood of original pixel. The BIM method is an iterative attack approach based on FGSM, where the pixel values are adjusted by α in each iteration. While it improves the efficiency in white-box attacks compared to FGSM, it is more computationally intensive.

Moosavi-Dezfooli et al. [31] presented the DeepFool method, which crafts perturbations based on the minimum distance between normal samples and the decision boundary of adversarial examples. After several iterations, the normal examples may cross the decision boundary, thereby achieving the attack’s objective of misclassifying the target model. Compared to the FGSM attack, the DeepFool attack requires adding only small perturbations to create highly effective adversarial examples. However, its drawback lies in the higher computational cost, as it requires gradually calculating the magnitude of the perturbations. Papernot et al. [32] introduced an effective attack method termed the Jacobian-based Saliency Map Attack (JSMA). Initially, the Jacobian matrix of the target model is obtained by calculating the forward derivative. Subsequently, the Jacobian matrix is utilized to construct the adversarial significance graph, reflecting the extent to which input features influence the output of the target network. Ultimately, the pixel with the highest adversarial significance value is selected to craft the perturbation.

### 2.2. Detection of Adversarial Examples

Adversarial example detection methods [33,34,35] are usually based on the following theory: given a class K neural network classifier, the original training set is $D={\left\{x_{i}\in R^{d}\right\}}_{i=1}^{N}$, and then we construct an adversarial sample set $D^{\prime }={\left\{x_{j}^{\prime }\in R^{d}\right\}}_{j=1}^{N}$ and design a detection method to distinguish $D^{\prime }$ and *D*. In recent years, there has been a lot of meaningful research in the field of adversarial defense: GAN was used to defend against adversarial attacks in [36]; autoencoder was employed for adversarial detection [37]; and diffusion model for adversarial purification [38]. Currently, methods for detecting adversarial examples predominantly encompass those based on data feature transformation, adversarial feature distribution statistics, and neural network characteristics. Methods based on data feature transformation typically investigate the discrepancies between normal and adversarial examples through noise reduction and reconstruction of input data. These methods implement adversarial example detection via thresholding or prediction inconsistency analysis. Methods based on adversarial feature statistics construct detectors by quantifying the example’s features at various levels. Detection methods leveraging neural network characteristics primarily build detectors by monitoring the example’s performance or behavior within the neural network, thus utilizing the network’s inherent properties [39,40,41].

For data feature transformation-based methods, Xu et al. [24] proposed two feature compression methods. They pointed out that CNNs do not require a large input feature space, and a larger feature space makes it easier for malicious attackers to generate adversarial examples. To address this, feature squeezing techniques, such as reducing image color depth and applying smoothing, are employed to create squeezed images from the initial input. Both the squeezed and original images are then individually predicted. AEs are detected by measuring the distance between the prediction vectors of the original input and each squeezed image. If any of these distances exceed a predefined threshold, the original input is classified as adversarial. While this method achieves good detection success rates against various adversarial attacks, its effectiveness relies heavily on the quality of the feature squeezing module, and setting the hyperparameter threshold remains a challenge. Gupta et al. [42] developed a CIIDefence method for the reconstruction of image regions that most significantly influence the present classification outcomes. Then, the adversarial examples are identified through analyzing the classification results of the original and reconstructed examples. CIIDefence does not require retraining or modification of the classifier. Liang et al. [43] proposed scalar quantization and smooth spatial filtering to lower noise. Using image entropy as the metric, the adaptive noise reduction in different types of images is performed. The adversarial examples are detected through judging the change in the prediction label of the examples before and after denoising. This defense method can protect against such as FGSM, C&W, and other adversarial attacks, but against larger adversarial disturbance, the defense performance is poor.

In the context of adversarial feature distribution statistics-based methods, Hendrycks et al. [44] observed differences in the variance and softmax distribution of the input coefficients after principal component whitening when comparing normal examples with adversarial examples as revealed by statistical analysis. Ma et al. [29] suggested using Local Intrinsic Dimensionality for characterizing the internal dimensions of disturbed regions, and to distinguish adversarial examples by analyzing the distance distribution among examples and their neighbors. Zhang et al. [45] developed an adversarial example detection method that leverages label sequence differences. The algorithm employs a reference value to mask the pixels and utilizes a random forest classifier to compute label sequences and LSD (label sequence difference) score for both the original and transformed examples. Then, these metrics are applied to train the classifier to identify adversarial examples. Feinman et al. [46] investigated the disparities in data distribution subspaces between normal and adversarial examples, applying kernel density estimation to identify adversarial examples distant from a manifold, and Bayesian uncertainty estimation to recognize adversarial samples in proximity to a manifold. These approaches highlight the diverse strategies in detecting subtle manipulations in data, essential for bolstering the robustness of machine learning models against adversarial threats. However, there is also a defect, that is, when the attacker understands the detection principle of the target model, they will adjust the attack strategy accordingly so as to bypass the detection mechanism and successfully attack.

Regarding methods based on neural network characteristics, Lu et al. [47] proposed the SafetyNet detection algorithm, where discrete codes derived from the input examples in the last ReLU layer are utilized as inputs for the support vector machine, aiming to construct an adversarial examples detector. Carrara et al. [48] encoded the activation position within the network for specific examples in the DNN feature space and proposed a feature distance space detection algorithm. The activation position of AEs deviates from the reference position of the normal example, enabling detection of the adversarial example according to the difference in the activation trajectory of the input example. This suite of approaches highlights the diversity in strategies employed to identify and counter adversarial attacks, leveraging the unique characteristics and behaviors within neural network models. Ma et al. [49] analyzed the internal structure of CNNs under various types of attacks and identified channels through which adversarial examples exploit vulnerabilities: the source channel and the activation distribution channel. They proposed a detection algorithm based on CNN channel invariants, known as Neural-network Invariant Checking (NIC). When testing input samples, NIC examines potentially adversarial inputs by running them through all invariant models to obtain independent predictions. These predictions indicate whether the input might cause a state that violates the invariant distribution. The final outcome of the NIC detection method is a joint decision based on these predictions. However, extracting invariants involves determining probability distributions, which requires a large number of models, leading to significant additional overhead. Additionally, the deployment of this detection framework is relatively complex.

Although current adversarial example detection methods can identify a subset of AEs generated by adversarial attacks, there are still some key issues to overcome. These problems include the low detection accuracy of adversarial examples, insufficient recognition ability of emerging adversarial examples, and how to eliminate adversarial interference. These issues underscore the need for ongoing research and development to enhance the robustness and adaptability of detection methodologies against a wider array of adversarial tactics. This paper focuses on these problems to enhance the detection accuracy of multi-class adversarial examples.

## 3. Adversarial Examples Detection Based on Frequency Domain Reconstruction

The fundamental principle of the proposed method is to consider disturbances as a form of noise leading to image distortion. Our strategy involves frequency domain reconstruction and gradient masking, aiming to significantly reduce the effects of adversarial noise. By introducing a subtle perturbation Δx to the input example *x* to obtain a new input $x^{\prime }$, the DNN model can give an incorrect output with high confidence, and $x^{\prime }$ here is called an adversarial example. The adversarial example $x^{\prime }$ satisfies $f\left(x^{\prime }\right)=f\left(x\right)$, and Δx≤ε, where $\varepsilon \in \;R^{n}$, and *n* denotes the dimension of the vector. The smaller the magnitude of the added perturbation, the less perceptible it becomes to the human eye. When $x_{ad\nu }$ is entered into a deep neural network (DNN), the output at each layer undergoes changes. To illustrate, taking the first layer as an example, $\varphi_{1}\left(x\right)=w_{1}\left(x\right)\cdot x+b_{1}$, the weights of this layer can be denoted as $w_{1}\left(x+\Delta x\right)=w_{1}\left(x\right)+\Delta w$. After inputting the adversarial examples, the representation of the neural network’s first layer becomes:

(5) $$\begin{array}{cc} \varphi_{1}\left(x_{ad\nu }\right) & =w_{1}\left(x+\Delta x\right)\cdot \left(x+\Delta x\right)+b_{1}\\ & =w_{1}\left(x\right)\cdot x+w_{1}\left(x\right)\cdot \Delta x+\Delta w\cdot x+\Delta w\cdot \Delta x+b_{1}\\ & =\varphi_{1}\left(x\right)+w_{1}\left(x\right)\cdot \Delta x+\Delta w\cdot x+\Delta w\cdot \Delta x \end{array}$$

$\varphi_{1}\left(x_{ad\nu }\right)$ and $\varphi_{1}\left(x\right)$ represent the output of the first layer with the model being fed with original and adversarial examples, respectively. From the equation, it becomes clear that the output of this layer is influenced by the last three terms, which are collectively denoted as $\Delta \varphi_{1}\left(x,\Delta x\right)$. These minor changes become amplified during the forward propagation process between layers and are represented by the term:

(6) $$\begin{array}{cc} f\left(x_{ad\nu }\right) & =\varphi_{l}\circ \varphi_{l-1}\circ \cdots \circ \varphi_{2}\circ \varphi_{1}\left(x_{ad\nu }\right)\\ & =\varphi_{l}\circ \varphi_{l-1}\circ \cdots \circ \varphi_{2}\circ \left(\varphi_{1}\left(x\right)+\Delta \varphi_{1}\left(x,\Delta x\right)\right) \end{array}$$

Predicted labels $l_{ad\nu }=\arg\max_{\theta }f\left(x_{ad\nu }\right)\neq l$ are significantly influenced by these perturbations. Although initially subtle within the network, these perturbations become progressively magnified in the deeper layers as the network propagates, eventually leading to misclassification.

### 3.1. Analysis of Adversarial Examples in the Frequency Domain

Song et al. [50] identified that a predominant portion of adversarial perturbations either constitute high-frequency signals or are superimposed onto high-frequency signals. This distribution bias originates from the propensity of neural networks to overfit low-frequency signals during the training phase, rendering them susceptible to the deceptive nature of high-frequency signals inherent in adversarial examples. In contrast, Zhang et al. [23] advocated for a methodology that entails the extraction of high-frequency components from adversarial examples through the process of adversarial training. A Fourier transform [51] on the spatial dimensions generates global statistical information, which enhances the distinguishability of various global representations and simplifies their learning process. The current work introduces a novel Fourier transform-based learning mechanism to mitigate or attenuate adversarial perturbations.

**Figure 1:**
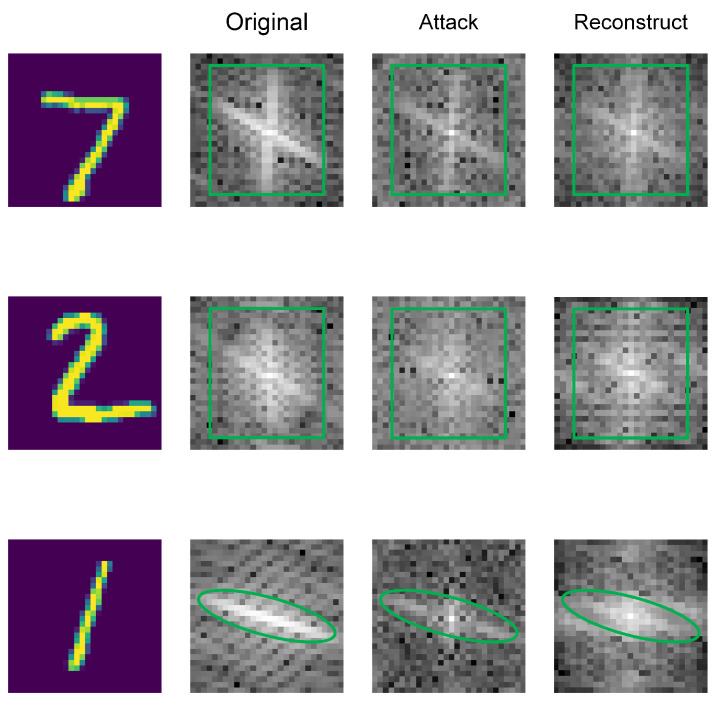
Comparison of frequency domain graphs of original example, adversarial example and reconstructed example. The first column is the spatial domain diagram of the original example, and the rest is the frequency domain diagram.

The physical significance of the Fourier transform is to convert the image’s grayscale distribution function to its frequency distribution function. In essence, performing a two-dimensional Fourier transform on an image to obtain a spectrum map is essentially a gradient distribution map of the image. The varying brightness of spots on the Fourier spectral map reflects the intensity of differences between a point in the image and its neighborhood, indicating the frequency magnitude at that point. Therefore, applying a Fourier transform to features provides an effective space for filtering out high-frequency perturbations and suppressing low-frequency signals, aiding in the reconstruction of clean samples from adversarial ones. As shown in Figure 1, from the aspect of the frequency domain, adversarial attacks add noise in the opposite direction of the neural network gradient, resulting in more bright and dark spots. Convolutional filtering and decoding reconstruction filter out or weaken the adversarial perturbations on the image. Intuitively, after the adversarial perturbations are weakened or eliminated, the transition of bright and dark spots in the image’s frequency domain becomes smoother.

In an ideal scenario, if we could reconstruct the initial input f(m,n) from an adversarial sample g(m,n), adversarial samples would be immediately detectable. However, due to the absence of essential knowledge regarding the noise sign(∇J(θ,x,y)), this is quite challenging to achieve. Therefore, we aim to eliminate perturbations in a classification sense and reconstruct the original image. That is to say, we transform g(m,n) into a new image $f^{\prime }\left(m,n\right)$, and then we observe whether its predicted class $L\left(f^{\prime }\left(m,n\right)\right)$ matches $L\left(f\left(m,n\right)\right)$.

### 3.2. The Proposed Model

The Frequency Domain Reconstruction Network (FDR-Net) is elucidated in this section, encompassing its comprehensive framework, the mechanism for detecting adversarial samples, and the details of its implementation. The FDR-Net utilizes convolutional neural networks as the backbone architecture. We first encode the input image. After the encoding phase, Fast Fourier Transform is employed to convert the image’s grayscale distribution function into its frequency distribution function. Through convolution operations, Adversarial perturbations in the frequency domain are eliminated, and low-frequency perturbations are suppressed. In the decoding phase, the introduction of inverse Fourier Transform and multi-layer convolution operations converts the frequency domain features back into a standard image. AEs are detected through comparing the classification results of both the initial image and the image reconstructed from the frequency domain.

**Figure 2:**
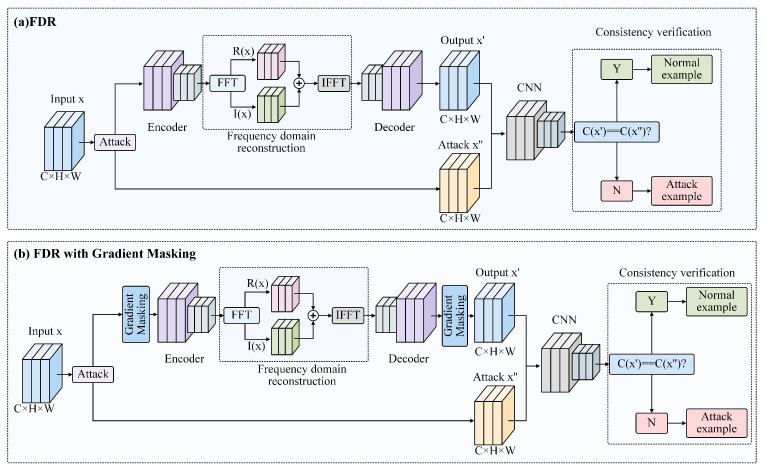
(**a**) The FDR structure first extracts the features of the input attack example, then performs Fourier transform to the frequency domain, and finally eliminates the attack in the frequency domain and reconstructs the original image. (**b**) The FDR with gradient masking structure first resizes the attack examples and extracts the features of the input attack examples, then performs Fourier transform to the frequency domain, and finally eliminates the attack in the frequency domain and reconstructs the original image before resizing to recover the input size.

The methodology put forward in this work primarily encompasses three components, frequency domain reconstruction, gradient masking, and consistency verification, as illustrated in Figure 2. In the frequency domain reconstruction phase, initially, the original examples are sampled to obtain frequency domain samples based on the Discrete Fourier Series. Subsequently, convolutional filtering and concatenation are performed on the amplitude and phase components of the frequency domain samples. Thereafter, the image is transformed from the frequency domain back into the spatial domain through inverse Fourier Transformation, followed by multi-layer convolutional local filtering for noise reduction. The filtered frequency domain examples are then reverse-normalized and mapped back to the RGB color space to obtain reconstructed examples. These reconstructed examples, along with the original adversarial examples, are inputted into the model to obtain classification results, which are then subjected to consistency verification.

### 3.3. Frequency Domain Reconstruction

The Fourier transform is indeed widely acknowledged for its utility in analyzing the frequency components of images. CNNs are particularly susceptible to the influence of high-frequency signals in images, which are often the carriers of misleading signals from adversarial examples. Moreover, the skewed distribution arises from the tendency of neural networks to fit low-frequency signals preferentially while insufficiently learning the distribution of high-frequency signals. Building upon the understanding, this study designed a frequency domain reconstruction mechanism to mitigate the vulnerability caused by high-frequency signals. In a Fourier spectral map, the varying brightness of the spots indicates the strength of the difference between a point in the image and its neighboring points, essentially representing the magnitude of the gradient. The low-frequency parts of an image correspond to areas with low gradients, while the high-frequency parts are the opposite. Larger gradients mean brighter intensity at a point, and smaller gradients mean weaker intensity. Given an image $x\in R^{H\times W\times C}$, the Fourier transform F(·) converts it to Fourier space, obtaining the complex component F(x), which is expressed as:

(7) $$F\left(x\right)\left(u,v\right)=\sum_{h=0}^{H-1}\sum_{w=0}^{W-1}x\left(h,w\right)e^{-j2\pi (\frac{h}{H}u+\frac{w}{W}v)}$$

Both the Fourier transform and its inverse procedure $F^{-1}\left(\cdot \right)$ can be effectively performed by FFT and IFFT algorithms. The amplitude component A(x)(u,v) and phase component P(x)(u,v) are expressed as:

(8) $$\begin{array}{cc} A(x)(u,v) & =\sqrt{R^{2}\left(x\right)\left(u,v\right)+I^{2}\left(x\right)\left(u,v\right)}\\ P(x)(u,v) & =\arctan\left[\frac{I(x)(u,v)}{R(x)(u,v)}\right] \end{array}$$

where R(x)(u,v) and I(x)(u,v) represent the real and imaginary operate, respectively. Consequently, A(x)(u,v) and P(x)(u,v) denote the amplitude and phase variations in the image frequency, respectively.

Secondly, we introduce a 1 × 1 convolutional layer to perform convolution operations on the real part R(x) and the imaginary part I(x), respectively, eliminating adversarial attacks in the frequency domain and ultimately obtaining new real part $R{\left(x\right)}^{\prime }$ and imaginary part $I{\left(x\right)}^{\prime }$:

(9) $$\begin{array}{cc} \; & R\left(x\right){\left(u,v\right)}^{\prime }=w_{1}\left(R\left(x\right)\left(u,v\right)\right)+b_{1}\\ \; & =w_{1}\left(A\left(x\right)\left(f\right)⊙\cos\left(P\left(x\right)\left(f\right)\right)\right)+b_{1}\\ \; & I\left(x\right){\left(u,v\right)}^{\prime }=w_{2}\left(I\left(x\right)\left(u,v\right)\right)+b_{2}\\ \; & =w_{2}\left(A\left(x\right)\left(f\right)⊙\sin\left(P\left(x\right)\left(f\right)\right)\right)+b_{2} \end{array}$$

Finally, we convert the processed Fourier domain feature $R{\left(x\right)}^{\prime }$ and $I{\left(x\right)}^{\prime }$ to their original space by employing the inverted Fourier transform:

(10) $$\begin{array}{c} x^{\prime }=F^{-1}\left(cat\left(R{\left(x\right)}^{\prime },I{\left(x\right)}^{\prime }\right)\right)\\ =\frac{1}{HW}\sum_{u=0}^{H-1}\sum_{v=0}^{W-1}F\left(u,v\right)e^{j2\pi (\frac{u}{H}h+\frac{v}{W}w)}\\ \hat{x}=conv\left(conv\left(conv\left(x^{\prime }\right)\right)\right) \end{array}$$

The inverse Fourier transform can be applied to the image features. Through decoding operations, they are transformed into a reconstructed image. Through a series of encoding, decoding, Fourier transformation, and convolutional filtering operations, the adversarial noise contained in the reconstructed image is reduced.

### 3.4. Gradient Masking

Adversarial attacks change the specific statistics of the input images, thus altering the model’s predictions. Mainstream adversarial attack algorithms seek out key pixels for minor perturbations that cause the classifier to make incorrect judgments. Thus, erasing these key pixels and reconstructing can reduce the perturbation’s effectiveness. In reality, adversarial perturbations have a specific structure. Therefore, we introduce the method of gradient masking, combined with Fourier transform, and design and experiment with image transformations that change these perturbation structures. Owing to the weak generalizability of iterative attacks, low-level image transformations such as resizing, padding, and compression might destroy the specific structure of adversarial perturbations, which can make them a good defense. If random transformations are applied, they can effectively defend against white-box attacks. The reason is that each image undergoes a random transformation, and the malicious attackers do not know the specific transformation during the process of generating adversarial noise.

Gradient masking employs a randomized layer to randomly adjust the input size, resizing original images of dimensions W×H×3 to a new size of $W^{\prime }\times H^{\prime }\times 3$. It is noteworthy that $\left|W^{\prime }-W\right|$ and $\left|H^{\prime }-H\right|$ need to be within a reasonably small range; otherwise, the performance on non-adversarial inputs can degrade obviously. The randomly resized image is then passed to the convolution part, followed by frequency domain reconstruction and decoding to yield the reconstructed image.

### 3.5. Consistency Check

After the above steps, the features reconstructed from the frequency domain are decoded and mapped back to the RGB color space, resulting in a filtered sample. Then, the reconstructed sample $x^{\prime }$ and the adversarial example $x^{\prime \prime }$ are input into the discriminator, yielding discrimination results $C(x^{\prime })$ and $C(x^{\prime \prime })$, respectively, where a consistency verification is performed (as shown in Figure 2). If the discrimination results are consistent $C\left(x^{\prime }\right)=C\left(x^{\prime \prime }\right)$, the example is judged to be normal $y_{x}=1$; otherwise, it is determined to be an adversarial sample $y_{x}=0$. The decision criterion can be mathematically represented as follows:

(11) $$y_{x}=\left\{\begin{array}{cc} 1 & C\left(x^{\prime }\right)=C\left(x^{\prime \prime }\right)\\ 0 & C\left(x^{\prime }\right)\neq C\left(x^{\prime \prime }\right) \end{array}\right.$$

## 4. Experimental Analysis

### 4.1. Experimental Setup

Hardware and Software Environment: The hardware configuration used in the current experiment consisted of an Intel Xeon E5-2673 v4 CPU and 8 NVIDIA GeForce RTX 2080Ti GPUs (with 256 GB memory and 8 GB VRAM). The experiment was conducted on the Ubuntu operating system, using Python 3.9 programming language and PyTorch 1.11 deep learning environment to implement all the detection methods. The convolution filter used 3 × 3 and 1 × 1 convolution kernel, the step length was 2, and the zero filling was adopted. The default function FFT in PyTorch was used for the fast Fourier transform. Axes and sequence of ints in FFT defaulted to −2 and −1. We adopted orthogonal normalization.

Datasets: In this study, we selected the MNIST, SVHN, and CIFAR-10 datasets, which are extensively recognized in the field of adversarial defense. The MNIST dataset refers to a collection of handwritten digit images with a training set of 60,000 samples and a test set of 10,000 samples. Each image is labeled into one of ten categories representing digits from 0 to 9, with each sample image having dimensions of 28 × 28 pixels. The SVHN dataset is a real-world image set of house numbers. It includes a training set with 73,257 samples and a test set with 26,032 samples. Each image is represented by pixel information at a resolution of 32 × 32 pixels. The CIFAR-10 dataset consists of color images categorized into ten classes. It comprises a total of 60,000 images with each class containing approximately equal samples (6000 per class). There are separate sets for training (50k) and testing (10k) purposes. The CIFAR-10 images have dimensions of size 32 × 32 pixels. These datasets provide diverse representations suitable for evaluating adversarial defenses, as they cover various everyday life categories such as aviation objects, vehicles, birds, cats, dogs, fish, horses and ships. The use of colored imagery in the CIFAR-10 dataset accurately reflects the real-world objects’ appearance, making it more relevant for applications involving adversarial samples.

Network Settings: We train a deep convolutional neural network on the basis of Lenet, which consists of three parts: convolutional layer, pooling layer, and fully connected layer. Using each convolutional layer, a pooling operation is executed, utilizing either average pooling or max pooling. Upon training, the network demonstrates robust performance across three benchmark datasets, achieving accuracies of 99.14% on MNIST, 81.42% on CIFAR-10, and 92.68% on SVHN. For the optimization process, the Adam algorithm is employed, and the network’s learning is guided through minimizing of the cross-entropy loss function.

### 4.2. Adversarial Examples Generation

We select five attack algorithms for experiments on three datasets, and also select three detection algorithms for comparison. Our designed adversarial attacks fall into two categories: targeted or non-targeted attacks. Targeted attacks use two target classes: the next class (the index of the true class plus one) and the least likely class (the class with the lowest predicted probability by the model). These attacks cover various norm versions to ensure comprehensive and reliable evaluation. Specifically, FGSM refers to an L∞ attack, C&W and DeepFool are L2 attacks, BIM is on the basis of an L1 norm attack, and MJSMA is on the basis of an L0 norm attack.

We comprehensively evaluate the performance of targeted FDR detection methods against five mainstream adversarial attack algorithms. Table 1 details the specific parameter settings of these five attacks on three datasets and provides their attack success rates, number of iterations, and perturbation magnitudes. To facilitate the quantification and comparison of perturbation magnitudes, we normalize the pixel values of the images to the range $\left[0,1\right]$ and then measure the perturbation magnitudes using the L2 norm. As shown in Table 1, these mainstream attack algorithms keep a balance between maintaining high attack success rates and controlling perturbation magnitudes at a relatively low level, achieving a trade-off between attack effectiveness and visual quality. ASR is the success rate of adversarial attack, and PSNR is the logarithm of the mean square error between clean samples and adversarial samples relative to the square of the signal maximum. When the disturbance values of FGSM attacks are 0.3, 0.1, and 0.3, respectively, the attack success rate is the highest on the three datasets. When the disturbance value of BIM attack is 0.3, the best attack success rate can be achieved on each dataset. When the iterations of C&W, DeepFool and MJSMA are 100,10 and 100, respectively, ASR reaches the best rate. At the same time, the PSNR values have little difference, and the image quality is stable. It should be noted that the score of PSNR cannot completely conform to the visual quality found by the human eye since the sensitivity of human vision to error is not absolute. When the attack is carried out, the unified attack parameters and disturbance amplitude can make the evaluation result more objective.

### 4.3. Detection of Adversarial Examples

**Figure 3:**
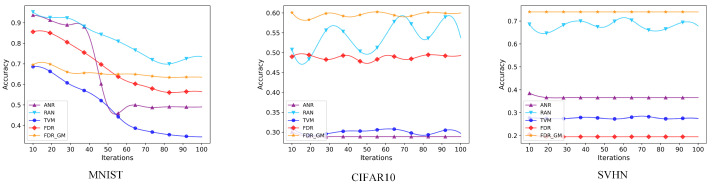
We select MJSMA attack algorithms to conduct experiments on three datasets and the number of iterations is 10–100.

We select five attack algorithms on three datasets and three detection algorithms for comparison. From the Table 2, we can see that under the attacks of FGSM, DeepFool, CW, and BIM, our FDR and FDR GM method maintains good results on the MNIST dataset. Under the FGSM attack, the accuracy of the proposed method is more than 5% higher than that of ANR method [43]. ANR uses smooth spatial filtering to denoise, but adversarial interference mostly exists in the frequency domain of the image. Both our FDR and FDR GM methods denoise in the frequency domain and use convolutional kernel for denoising, so the detection accuracy is better. Under the DeepFool attack, the accuracy of the proposed method is more than 30% higher than that of the RAN method [52]. RAN uses random resizing and random padding to mitigate the adversarial effect. The method has a limited effect on frequency domain interference, so the detection accuracy is lower than the method proposed in this paper. Among all the attack methods, the accuracy of the detection method in this paper is higher than that of TVM [53]. The reason may be that TVM uses Bernoulli random variables to reconstruct some pixels. This Bernoulli randomization process loses some feature information. Under the MJSMA attack, the detection algorithm RAM is more advantageous. MJSMA uses an adversarial salience map for its attack, while the picture in the MNIST dataset is a grayscale map. Therefore, the position of the pixels of the two points with the largest absolute values found by the MJSMA attack may change greatly, and the RAN uses random resizing to mitigate the antagonistic effect. This process easily removes some of the disturbance points in MJSMA. However, for the MJSMA attack as shown in Figure 3, we compare all the detection algorithms on three datasets together. The images from the CIFAR10 and SVHN datasets are three-channel color RGB images. The FDR GM algorithm has advantages over the RAM detection algorithm on the CIFAR-10 and SVHN datasets, and its defense performance is more stable. Our FDR GM method performs well on the other two datasets, CIFAR10 and SVHN, indicating that FDR GM is more suitable for images with higher information content, such as RGB images. In contrast, the FDR method is more applicable to images with lower information content, like the MNIST dataset. We speculate that this is because the GM module may lose some information, leading to poorer performance on single-channel datasets. On the other hand, RGB datasets contain richer information content, which can be utilized by the GM module. Furthermore, we can observe that our proposed FDR and FDR GM algorithms outperform the three compared algorithms overall, achieving a maximum defense success rate of 93.58% against the CW attack. This demonstrates that our proposed methods can effectively defend against attack algorithms across different datasets.

**Figure 4:**
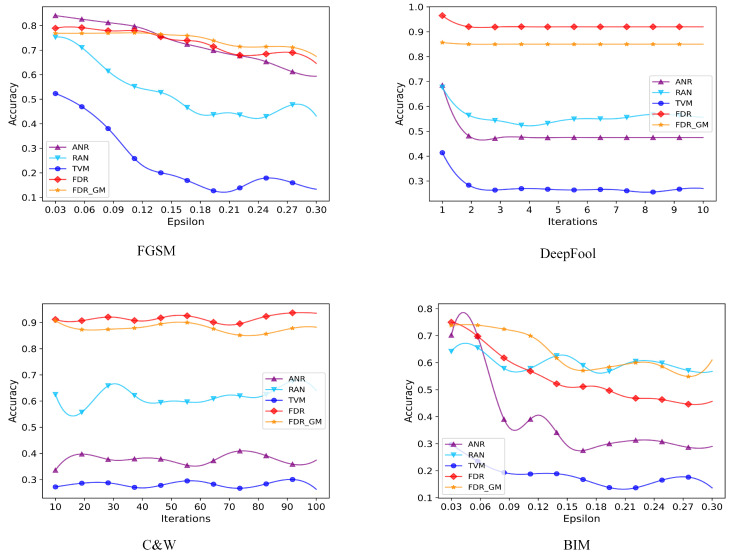
We select four attack algorithms to conduct experiments on the MNIST dataset and the number of iterations of DeepFool and C&W attack algorithm is 10 or 100, respectively. We compare the proposed algorithm with 3 different detection algorithms.

**Figure 5:**
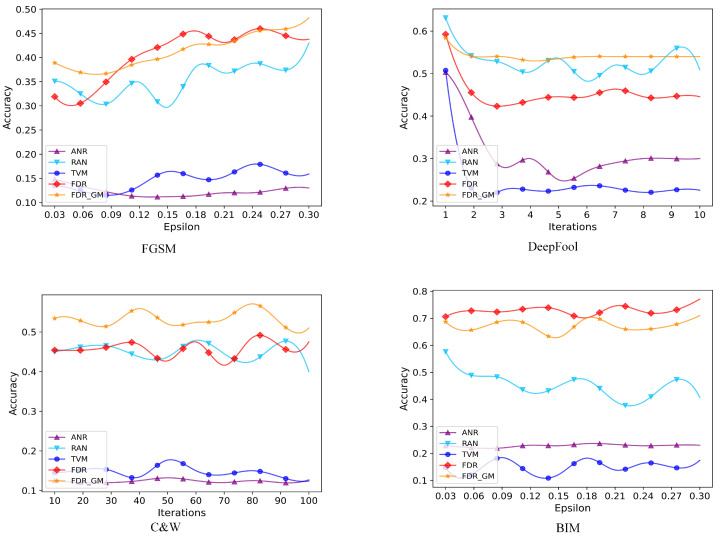
We select four attack algorithms to conduct experiments on the CIFAR10 dataset, and the disturbance value of the FGSM and BIM attack algorithms is 0.03–0.30. We compare the proposed algorithm with 3 different detection algorithms.

**Figure 6:**
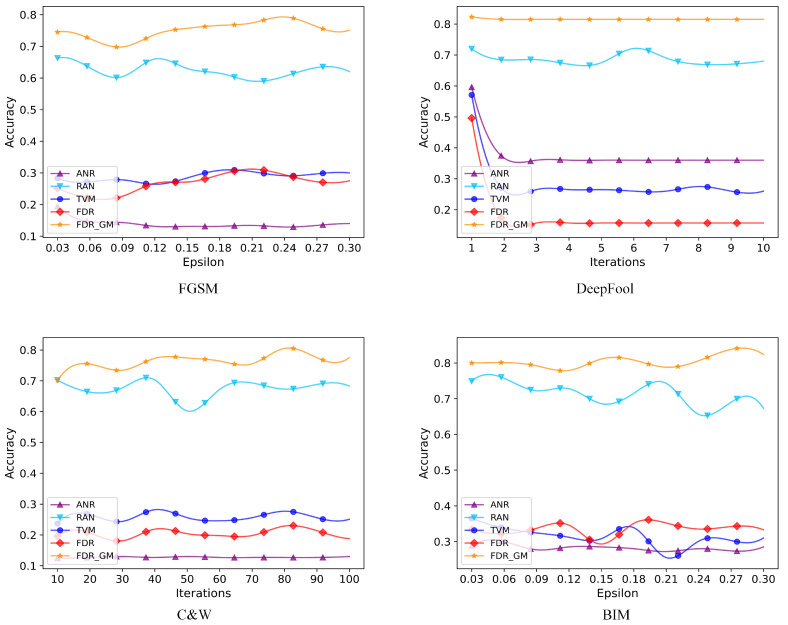
We select four attack algorithms to conduct experiments on the SVHN dataset, and the disturbance value of the FGSM and BIM attack algorithms is 0.03–0.30. We compare the proposed algorithm with 3 different detection algorithms.

With the purpose of more intuitively demonstrating the impact of our proposed algorithm, we perform model attacks on three datasets using attack algorithms of four different methods. According to the optimal interference value and number of iterations calculated by us in the previous section, the number of iterations for the DeepFool attack algorithm increases from 1 to 10. The number of iterations for the C&W attack algorithm increases from 10 to 100. The disturbance e value increases from 0.03 to 0.30. The accuracy curve of our proposed method FDR GM and the other three defense algorithms are illustrated in Figure 4, Figure 5 and Figure 6. From the figure, we can see that the defense effect of our proposed method under the attack algorithm with different iterations is generally better than that of other defense algorithms, which is the same as the analysis of experimental results in Table 2. On the MNIST dataset, ANR and RAN detection algorithms sometimes perform slightly better than our algorithms under FGSM and BIM attacks. But overall, the performance of our algorithm is more stable. And for different e values, the average detection accuracy is higher. On the other two datasets, sometimes some algorithms perform better. However, our algorithm has more robust performance and smaller average detection variance under different attack algorithms. On the CIFAR10 and SVHN datasets, FDR GM performs better than FDR. It shows that for three-channel RGB images with richer information content and greater gradient variation, adding gradient masking is more beneficial to eliminate the adversarial disturbance.

**Figure 7:**
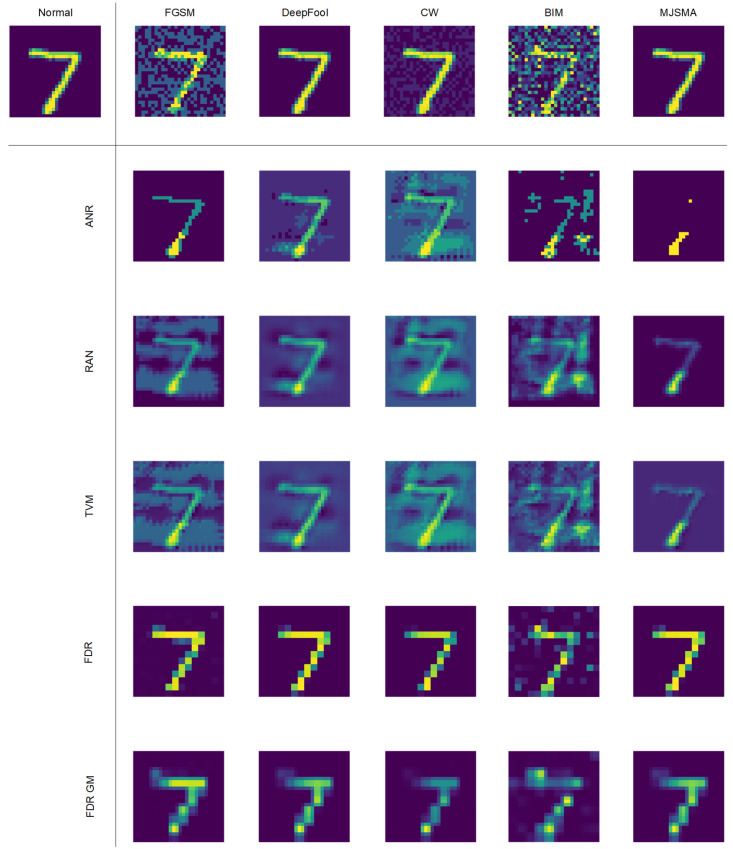
We visualize the recovery results of FDR GM method and other attack example detection methods under different attack algorithms.

Meanwhile, to more intuitively show the effect difference, we visualize the attack samples after the recovery of the detection algorithm in Figure 7. Clearly, the figure shows that our proposed FDR is closer to the original sample and contains less attack noise. The proposed FDR GM algorithm can maintain the rough outline of the original sample and filter out almost all noise. The goal of the adversarial defense is to detect AEs and maintain the stability of the classification network. Compared with the other three detection algorithms, the proposed algorithm balances the detection accuracy and the sample fidelity after filtering under five kinds of attacks.

To assess the impact of each module in our proposed FDR GM method, we select five attack algorithms for ablation experiments on three datasets and compare them with different modes. The experimental results are presented in Table 3, Table 4 and Table 5. Based on Table 3, it can be seen that under the five attacks, the combination of Conv and GM modules has more advantages in terms of average detection accuracy and variance. The detection robustness of the combination model is stronger. Under DeepFool and C&W attacks, the detection accuracy of the Conv module is better than the other two models. But the advantage is small when comparing 15 sets of data. The reason for this advantage may be that the GM module could lose information from low-resolution grayscale images. And because MNIST has only digital recognition, limited interference can make the recognition wrong. This also makes the Conv denoising module have a large role space. In Table 4, the overall participation of the Conv and GM modules achieves better detection results. The Conv filter module performs 6% better under BIM attacks. But considering the real-world deployment of detection algorithms, it is impossible for adversaries to adopt only one attack method. Therefore, the combined use of Conv and GM modules brings higher defense benefits. As shown in Table 5, the combined use of Conv and GM modules achieves better detection accuracy than other combinations in the SVHN dataset. Compared with the Conv module, the GM module achieves better detection accuracy. This may be because SVHN is a house number in the real street view, with a more complex background and more diverse number variations. So Conv filtering is of limited use. Finally, we find that the combination of Conv and the GM module can effectively deal with various adversarial attacks under complex images and achieve a good defense effect.

## 5. Conclusions

With the purpose of improving the capability of the CNNs classification network to defend against AEs, this study puts forward a novel detection method based on frequency domain reconstruction, which is universal to all kinds of attack methods. In the process of detection, Fourier transform is used to transform the image from the spatial domain to the frequency domain, and the convolutional neural network is used to filter and denoise the frequency domain. The gradient masking algorithm is used for multi-feature input. The adversarial examples are detected by the classification consistency of the reconstructed examples and the original samples by the deep convolutional neural network. Five attack algorithms and three detection algorithms are compared on three datasets. Numerous experiments prove that the proposed FDR GM and FDR method in this study has a high detection accuracy. The proposed method does not need to modify the original network and has the advantage of simple application. Relative to other defense methods, the proposed method has a strong generalization ability among models and can defend against attacks against samples across models, effectively ensuring the credibility of the classification results. In future research, we will also focus on exploring integrated robustness enhancement methods for detection models and target models to lower the likelihood of attackers spoofing target classifiers and adversarial detection models. Meanwhile, the adversarial example detection method proposed in the current work can be transferred to other fields to detect adversarial examples, such as detecting adversarial examples in remote sensing image detection, video processing, and natural language processing.

## Figures and Tables

**Table 1:** Comparison of adversarial attack performance.

Attack	Parameters Description	Dataset	Parameter Settings	ASR	PSNR
			ε = 0.1	34.42	70.74
		MNIST	ε = 0.2	84.49	64.80
			ε = 0.3	95.23	61.34
			ε = 0.1	95.59	68.31
FGSM	ε: perturbation	CIFAR10	ε = 0.2	93.94	62.50
			ε = 0.3	92.00	59.27
			ε = 0.1	80.00	68.23
		SVHN	ε = 0.2	84.69	62.39
			ε = 0.3	86.53	59.14
			max_ite = 1	64.06	74.02
		MNIST	max_ite = 10	99.99	68.04
			max_ite = 50	99.99	68.04
			max_ite = 1	33.59	96.28
DeepFool	max_ite: attack iteration	CIFAR10	max_ite = 10	99.99	89.56
			max_ite = 50	99.99	89.56
			max_ite = 1	17.53	91.95
		SVHN	max_ite = 10	99.99	84.50
			max_ite = 50	99.99	84.50
			max_ite = 10	93.24	62.79
		MNIST	max_ite = 100	99.99	63.27
			max_ite = 1000	99.99	63.24
			max_ite = 10	98.48	63.27
C&W	max_ite: attack iteration	CIFAR10	max_ite = 100	99.99	63.19
			max_ite = 1000	99.99	63.19
			max_ite = 10	98.77	63.05
		SVHN	max_ite = 100	99.79	62.96
			max_ite = 1000	99.99	62.61
			ε = 0.1	86.31	59.03
		MNIST	ε = 0.2	89.55	54.43
			ε = 0.3	90.11	50.44
			ε = 0.1	89.59	60.58
BIM	ε: perturbation	CIFAR10	ε = 0.2	89.53	56.86
			ε = 0.3	89.72	55.80
			ε = 0.1	89.49	60.21
		SVHN	ε = 0.2	89.62	56.86
			ε = 0.3	89.72	55.80
			max_ite = 10	98.29	79.03
		MNIST	max_ite = 100	99.99	65.39
			max_ite = 1000	99.99	65.39
			max_ite = 10	99.12	75.18
MJSMA	max_ite: attack iteration	CIFAR10	max_ite = 100	99.99	75.70
			max_ite = 1000	99.99	75.70
			max_ite = 10	99.87	74.54
		SVHN	max_ite = 100	99.99	73.83
			max_ite = 1000	99.99	73.83

**Table 2:** Comparison of detection accuracy results on three datasets.

		Attack Methods				
**Dataset**	**Method**	**FGSM**	**DeepFool**	**C&W**	**BIM**	**MJSMA**
	ANR	59.36	47.50	37.44	29.01	49.07
	RAN	43.17	55.66	64.25	56.78	73.51
MNIST	TVM	13.33	27.02	26.32	13.65	34.48
	FDR	64.63	92.46	93.58	45.70	56.43
	FDR GM	67.47	85.43	88.23	61.08	63.51
	ANR	13.27	30.39	12.75	23.24	28.86
	RAN	43.21	50.93	40.70	40.55	53.82
CIFAR10	TVM	15.91	22.55	12.42	17.69	29.73
	FDR	43.87	44.55	47.58	77.05	49.36
	FDR GM	48.24	54.08	51.05	71.15	60.39
	ANR	14.29	36.64	13.08	28.57	36.64
	RAN	62.76	68.63	68.39	67.30	67.91
SVHN	TVM	29.94	26.62	25.13	31.03	27.52
	FDR	27.52	15.70	18.80	33.30	19.53
	FDR GM	75.18	81.55	77.52	82.42	74.06

**Table 3:** Comparison of ablation results of FDR GM methods under different attack algorithms in the MNIST dataset.

Conv	GM	FGSM	DeepFool	C&W	BIM	MJSMA
✓		64.63	92.46	93.58	45.70	56.43
	✓	38.54	54.85	68.59	27.48	37.59
✓	✓	67.47	85.43	88.23	61.08	63.51

**Table 4:** Comparison of ablation results of FDR GM methods under different attack algorithms in the CIFAR10 dataset.

Conv	GM	FGSM	DeepFool	C&W	BIM	MJSMA
✓		43.87	44.55	47.58	77.05	49.36
	✓	27.48	37.59	18.65	25.86	37.85
✓	✓	48.24	54.08	51.05	71.15	61.39

**Table 5:** Experimental results of ablation under different attack algorithms in the SVHN dataset.

Conv	GM	FGSM	DeepFool	C&W	BIM	MJSMA
✓		27.52	15.70	18.80	33.30	19.53
	✓	48.74	58.65	46.47	58.52	56.57
✓	✓	75.18	81.55	77.52	82.42	74.06

## Data Availability

The datasets applied in this paper are all open-source datasets. The open source URLs are shown below. https://yann.lecun.com/exdb/mnist/, https://www.cs.toronto.edu/~kriz/cifar.html, http://ufldl.stanford.edu/housenumbers/, accessed on 22 August 2024.
